# The PSIPRED Protein Analysis Workbench: 20 years on

**DOI:** 10.1093/nar/gkz297

**Published:** 2019-04-26

**Authors:** Daniel W A Buchan, David T Jones

**Affiliations:** UCL Bioinformatics Group, Department of Computer Science, University College London, Gower Street, London WC1E 6BT, UK

## Abstract

The PSIPRED Workbench is a web server offering a range of predictive methods to the bioscience community for 20 years. Here, we present the work we have completed to update the PSIPRED Protein Analysis Workbench and make it ready for the next 20 years. The main focus of our recent website upgrade work has been the acceleration of analyses in the face of increasing protein sequence database size. We additionally discuss any new software, the new hardware infrastructure, our webservices and web site. Lastly we survey updates to some of the key predictive algorithms available through our website.

## INTRODUCTION

Today there are a great number of web services available covering all aspects of research in the Life Sciences. In biochemistry, important resources hosting primary data are hosted by the NCBI, EBI and RCSB (1–3). Additionally a number of labs around the world have provided invaluable second tier analysis services to the research community for a number of years. Examples include PHYRE, MPI Bioinformatics Toolkit and the Expasy: SIB Bioinformatics Portal (4–7).

The PSIPRED Protein Analysis Workbench is a world renowned web service providing a diverse suite of protein prediction and annotation tools focussed principally on structural annotations of proteins. The service has been in near-continuous operation for 20 years. The service runs around 250 000 predictions for users each year. In the following paper we describe work we have completed to bring our web services in to the modern web era. Most importantly the implementation of a novel workflow management platform which allows us to easily deploy new predictive tools to our website.

Workflow Management Systems solve the generic problem of turning chains of data processing steps into repeatable or automated processes. Typically, a given data analysis can be decomposed into a series of data transformation steps, where any given step depends on the outputs of the previous step or steps. The complete data analysis task can then be defined by the input data, the required outputs and the dependency graph of data transformations that take the data from input to output. Often, data analysis tasks decomposed into steps in this manner are regarded as pipelines or workflows. A range of workflow management tools, both proprietary and open source, are readily available. Such as Microsoft Azure, Spotify's Luigi, Twitter Storm, HADOOP and so forth. In the Open Source space Apache Taverna (https://taverna.incubator.apache.org/) is a user-centric tool for specify workflows which can access a heterogenous set of data processing web services. Common Workflow Language (CWL) (8) is a specification for describing workflows to ensure they are portable across hardware and execution platforms. Several implementations capable of executing CWL workflows are available.

Data analysis webservices in the biosciences face many similar challenges to one another. High throughput data generation technologies continue to increase the pace that new data is deposited in public databases. Genbank and the PDB have shown near exponential growth over the previous 20–40 years with no signs of slowing down (1,2). Researchers have also pioneered the use of increasingly computationally intensive algorithms or predictors ranging from dynamic programming (9) to deep neural networks in the present day. Without algorithmic or hardware advancements database search and algorithmic run times will lengthen. These provide challenges to web services which wish to ensure the services offered run in a timely fashion.

### Web site developments

Since 2015, we have undertaken to completely rewrite all the code which serves and runs the PSIPRED Workbench site. Our principle goal was to greatly reduce prediction runtimes for users. The expanding size of sequence data banks such as UniRef and GenBank has greatly increased the runtime of software such as PSIBLAST in the intervening years. We also wished to ensure the website will be able to cope with the increasing resource and computational demands of running a contemporary bioscience web site.

#### New web design

**Figure 1.**
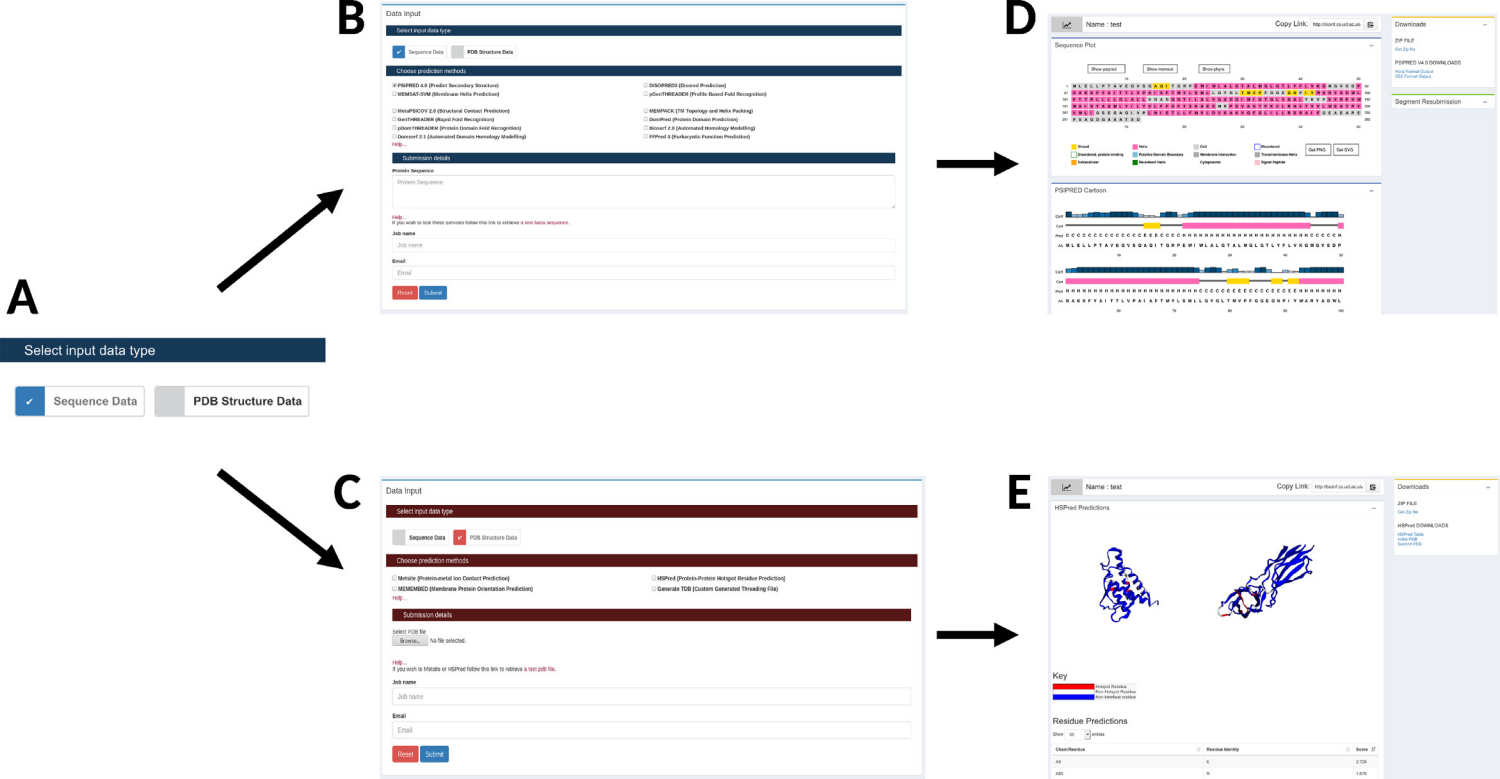
The new website layout. (**A**) Users select which kind of data they have to analyse, either protein Sequence data or a PDB file. (**B**) Users are presented with the set of 12 sequence analysis algorithms available on the site. Sequence analysis jobs present users with the sequence results page (**D**), the main panel shows the sequence annotation map and further results and tables are presented below. On the right hand side, a panel to download results files and to resubmit sequence segments is shown. For PDB analyses (**C**), users are presented with the four PDB structure analysis tools. PDB results (**E**), typically show the predictions annotated on the PDB file that was submitted, again results files can be downloaded from the panel on the right.

A major part of the work we completed is the web site's redesign (see Figure 1). This was completed to ensure all our predictive methods were more easily accessible in a single location and that the results produced were more intuitively laid out. The new site makes use of modern web frameworks including bootstrap (https://getbootstrap.com), Ractive (https://ractive.js.org) and d3 (https://d3js.org/). We have also moved all processing of server results and diagram rendering to the users's web browser, this reduces the processing load on our backend servers allowing us to calculate a greater number of concurrent users’ analyses.

The input form remains much as it was previously. The principle difference is that users can now select whether their input data is protein sequence or PDB data (see Figure 1, part A) and the form will present the appropriate predictive methods (see Figure 1B and C) there are 12 sequence analysis methods and four protein structure analysis methods.

**Figure 2.**
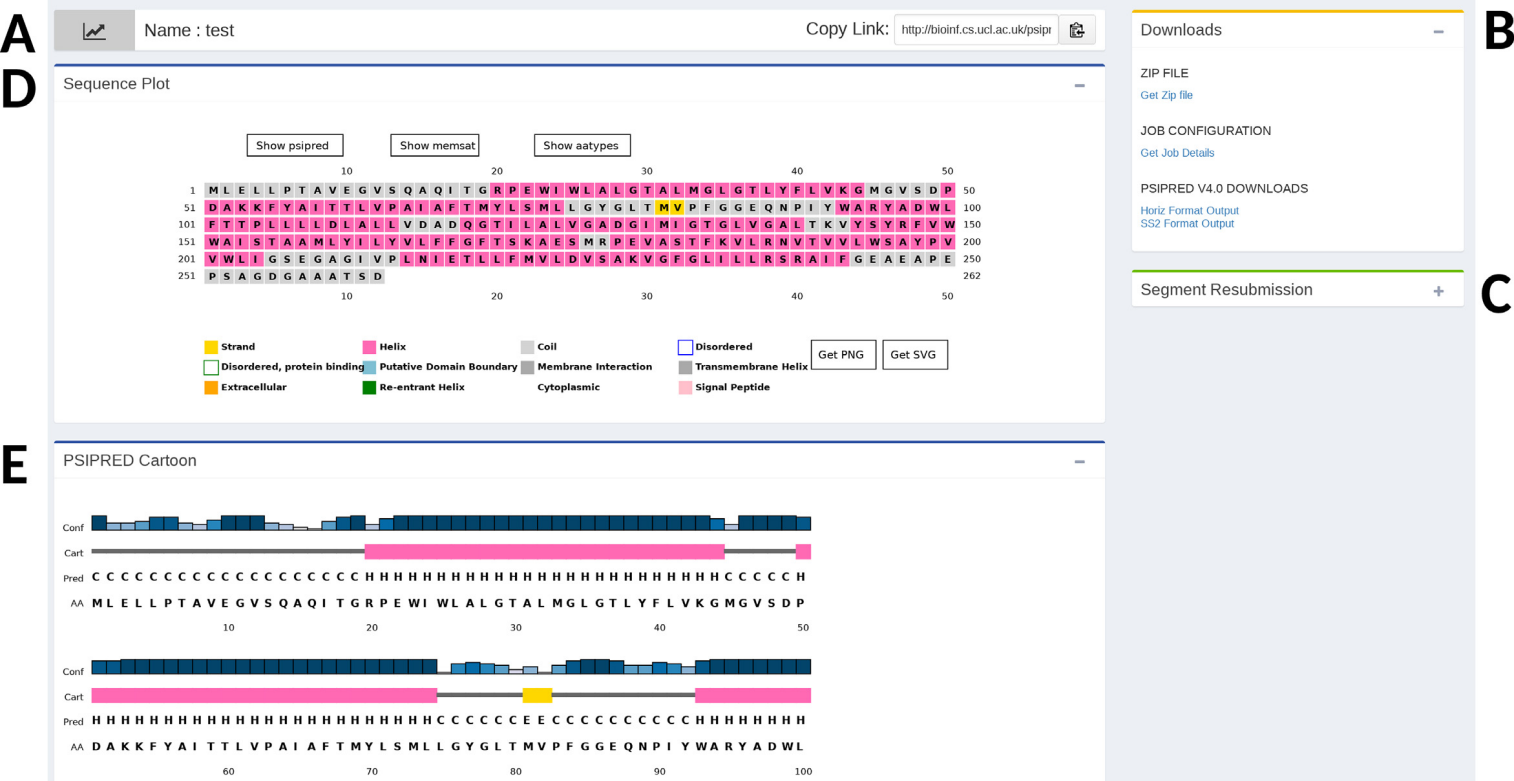
The new results presentation. Area **A** is the header section returning the submission name the user provided and the ability to copy the results page URL. Area **B** contains the file Download panel which are either available individually or as a zip file. Area **C** holds the Segment Resubmission panel which lets users select a sub-segment of the query sequence to re-submit to the server. Area **D** is the Sequence Annotation Plot. This shows the user's query sequence and any predictions annotated on it, in this instance it shows a PSIPRED secondary structure prediction. The sequence annotation panel is not displayed for structure prediction results. Areal **E** shows one or more further results panels, in this case it shows the PSIPRED results cartoon.

Upon protein sequence submission the results page has been updated (Figure 1D and Figure 2). On the right-hand side we show three panels: an estimate of the runtime for the analysis, a panel that holds links to the results file downloads (Figure 2B) and a panel which holds the resubmission widget (Figure 2C). For the results files users can download the files of interest or download a zip file of all the results files. The resubmission panel allows users to select some sub-region of their query sequence, using the linear start-stop coordinates of the sequence. This sub-region can then be submitted to the server for further analysis. Users can also follow the ‘Help & Tutorials’ link on the page for further details about the methods and using the server.

The principle change to the website is the presentation of the results (Figure 2). The results page is now divided in to five main sections. The first section (Figure 2A) is a title banner which holds the name of the job the user selected and a small widget allowing the user to easily copy the results page URL to the desktop clipboard. In the Downloads section (Figure 2B) users can access the individual results files used to prepare the results page and visualizations. We also provide a link to a ‘Job Configuration’ file which details the analysis steps performed to make the prediction, this contains software and dataset versioning information. All these files are also available as a zip file. Below the downloads section (Figure 2C) is the Segment Resubmission widget, this allows users to select an arbitrary sub-segment of their query sequence and resubmit it to one of the sequence prediction methods. Users may find this useful in cases when results, such as those from DISOPRED, DomSerf, DOMPRED or pDomTHREADER, indicate the query sequence has discrete domains or regions that they wish to perform further analysis on. For sequence results, the Sequence Plot panel (Figure 2D) shows any predictions made, annotated on to the query sequence. This panel is omitted when PDB files are submitted for analysis. Lastly the results area (Figure 2E), this area holds one or more panels which display alternative or ancillary results information. These take the form of additional diagrams, charts and graphs or results tables depending on the predictive method.

#### Social media & support

To make it easier for users to contact us and get real time updates about the PSIPRED webserver, we have also recently launched our new Twitter account, https://twitter.com/psipred. This account disseminates up to date information about the current state of the server and any issues, updates, downtime notices and relevant news. Users are welcome to request help, ask questions or give feedback via this service. This service now sits alongside our contact email, psipred@cs.ucl.ac.uk.

### Software & algorithm improvements

The PSIPRED Protein Analysis Workbench offers a total of 16 protein analysis methods (see Table 1). Since the publication or our previous server paper the majority of these remain as previously published (10). In this section, we give a brief overview of only those methods that have seen significant improvements since our 2013 publication, namely: PSIPRED, MetaPSICOV, FFPred, DISOPRED, Bioserf & Domserf. Readers interested in our other methods and their current performance should consult the references listed in Table 1.

**Table 1.** Methods available via the PSIPRED workbench

Method	Input	Summary	Citation
**PSIPRED 4.0**	Protein Sequence	Secondary structure prediction	Protein secondary structure prediction based on position-specific scoring matrices (11)
**DISOPRED3**	Protein Sequence	Disordered residue prediction	DISOPRED3: precise disordered region predictions with annotated protein-binding activity (12)
**MEMSAT-SVM**	Protein Sequence	Membrane helix prediction	Predicting transmembrane helix packing arrangements using residue contacts and a force-directed algorithm (13)
**GenTHREADER, pGenTHREADER & pDomTHREADER**	Protein Sequence	Fold recognition	pGenTHREADER and pDomTHREADER: new methods for improved protein fold recognition and superfamily discrimination (14)
**MetaPSICOV 2.0**	Protein Sequence	Structural contact prediction	MetaPSICOV: combining coevolution methods for accurate prediction of contacts and long range hydrogen bonding in proteins (15)
**DomPred**	Protein Sequence	Protein domain boundary prediction	Computer-assisted protein domain boundary prediction using the DomPred server (16)
**BioSerf 2 & DomSerf 2.1**	Protein Sequence	Automated homology modelling pipeline	Scalable web services for the PSIPRED Protein Analysis Workbench (10)
**FFPred3**	Protein Sequence	GO Term functional prediction	FFPred 3: feature-based function prediction for all Gene Ontology domains (17)
**MEMPACK**	Protein Sequence	Estimates packing and orientation of membrane helices	The MEMPACK alpha-helical transmembrane protein structure prediction server (18)
**Metsite**	Protein Structure	Metal binding site prediction	Predicting metal-binding site residues in low-resolution structural mode (19)
**HSPred**	Protein Structure	Protein-protein interaction hotspot prediction	Predictions of hot spot residues at protein-protein interfaces using support vector machines (20)
**MEMEMBED**	Protein Structure	Membrane protein orientation prediction	Membrane protein orientation and refinement using a knowledge-based statistical potential (21)
**Generate TDB**	Protein Structure	Creates custom TDB file for use with genthreader, pGenTHREADER or pDomTHREADER	pGenTHREADER and pDomTHREADER: new methods for improved protein fold recognition and superfamily discrimination (14)

#### PSIPRED 4

PSIPRED is a popular and cutting edge protein secondary structure method. This remains the most popular method on our server. Since the previous publication of the webserver substantial work on the PSIPRED method has been completed. Our webserver now offers PSIPRED version 4. PSIPRED 4 has implemented the first architectural change to PSIPRED since its first release in 1999. The main change is to a deeper neural network architecture with two hidden layers rather than just one, and with rectifier activations rather than sigmoid. The other change is that the input window has been extended from 15 residues to 33 thanks to using sparse connections between the input layer and first hidden layer. The latest version has a Q3 secondary structure prediction accuracy of 84.2%. A full benchmark and comparison with equivalent methods will be the topic of a future publication.

The new PSIPRED web presentation updates the PSIPRED cartoon (Supplementary Information Figure 1). As previously the results are presented on four rows representing the confidence score (Conf), the assignment in cartoon form (Cart), the assignment in three state letters (Pred) and the query sequence (AA).

#### FFPRED 3

FFPRED3 is the latest update of the FFPred GO term prediction method. Algorithmically the method remains as previously described; a number of sequence feature predictors are run for the query sequence and then a library of SVMs representing individual GO terms is scanned with a prediction for each GO term produced (22). The new code base integrates a number of bug fixes and some major changes to make the code more portable. The principle update between versions 2 and 3 is the inclusion of a new library of Fly-specific GO terms. The library to be searched is selected when users select the FFPred method on the website's landing page. We currently support predictions for 868 human GO terms and 288 fly GO terms.

#### MetaPSICOV 2

MetaPSICOV 2 is the contact prediction pipeline we entered in 2016’s CASP community experiment, CASP12 (23). This meta-predictor integrates contact predictions from 3 coevolutionary contact prediction methods: PSICOV (24), MF-DCA/FreeContact (25) and CCMPred (26). The main improvements to the method are a novel domain finding algorithm and new neural network architecture for integrating the three contact predictions (Supplementary Information Figure 4 shows the data analysis pipeline that MetaPSICOV follows). We direct interested readers to the MetaPSICOV and MetaPSICOV2 papers (15,27). MetaPSICOV was among the best performing contact predictors in CASP12 with a mean precision of 43.27%.

#### DISOPRED 3

DISOPRED3 (12) is a substantial update to our previous DISOPRED2 method (28). This produces predictions by integrating three predictors, DISOPRED2, a new neural network predictor and a simpler nearest neighbor approach. A small neural network then combines the outputs of these. DISOPRED3 also now produces predictions for both disordered regions and detects disordered protein binding regions. It typically displays Precisions and MCC values at approaching double and often more than triple those of DISOPRED2 for all classes of disorder region prediction that were tested, i.e. for disordered regions binned at lengths no shorter than 4, 20, 30 and 40 amino acids and for internal disordered regions. DISOPRED3 does not substantially improve the quality of N and C terminal disorder predictions as DISOPRED2 was already a strong predictor over these regions.

#### DomSerf 2.1 & BioSerf 2

The DomSerf and BioSerf pipelines remain as previously published (10). Both pipelines perform a detailed homology search of the CATH database and/or PDB to find viable homology modeling templates and these templates and alignments are then used with MODELLER (29) to build a series of models from which the most representative model is returned to the user (see Supplementary Information, Figure 3). The principle changes for our automated homology modelling pipelines are that we have now made the code available to users for the first time (https://github.com/psipred/bioserf) and in doing so fixed a large number of previously unseen bugs. Previously ∼10% of runs of these pipelines were failing to produce models due to bugs and we expect most of such modelling jobs to complete.

### Backend server improvements

#### Hardware

To support the new website we have also substantially upgraded the hardware our software runs on. The previous web server architecture regularly hit its peak processing capacity, often leading to long queue times for jobs submitted by users. The previous web server had 40 cores and 160GB of memory available for data processing. Our new server greatly updates this capacity providing 192 modern cores with more than 1TB of RAM available. With these hardware changes alone, we anticipate that both queue times and runtimes will be greatly decreased for users. Preliminary analysis suggests that run times will be between 4 and 20 times faster than they were in the recent years.

#### Improved sequence search and results caching

Many of the predictors offered by our service, such as PSIPRED or FFPred (17), begin with a comprehensive protein database search using PSIBLAST (30). As the size of the underlying sequence datasets have grown, PSIBLAST runtimes have significantly increased. We typically saw runtimes from 20 to 80 min per target sequence. To address this issue, we have adapted the sequence searching steps in our algorithms to make use of HHBlits (31), this typically has shorter runtimes (around five times faster) and often <5 min. PSIBLAST is retained only to convert these sequence search results in to the required residue propensity matrices (PSSMs) which many of our methods require. Using HHBlits for sequence searching now also means that all our predictive methods can take a Multiple Sequence Alignment as input. This means that pGenTHREADER, MEMSAT-SVM, MEMPACK and pDomTHREADER are no longer restricted to single sequence inputs.

To accelerate jobs for users, we now cache any results generated by HHBlits. The BLAST PSSM cache (https://github.com/psipred/blast_cache) is a simple database backed web service. This service is part of a broader change to move the PSIPRED Workbench to a Service Oriented Architecture (SOA). When a new job is submitted and requires a sequence database search we now first query our cache of existing PSSMs. If a valid, pre-existing result is present, it is returned and the sequence search is skipped. These cached results expire after one month to ensure results for sequences don’t become fixed or stale. Alongside the PSSM we also cache comprehensive logging information about the sequence search. We would hope to use this information to better optimise our service in the future, for instance in understanding the distribution of organism proteins that users submit and to track the rate at which sequence search results change over time. Methods such as MEMEMBED or HSPred do not make use of the PSSM cache as their predictions do not start with a sequence database search, additionally these methods have short run times and result caching is less useful in these instances.

#### Workflow management engine

The main backend software development effort for the PSIPRED web server has been our novel Workflow Management Engine, Analytics Automated (manuscript in preparation). Our previous code base prevented us from adding new predictive methods to the web site in a timely fashion. The majority of our engineering effort for the new web server has been focussed on replacing the old backend code to eliminate this issue. Our new workflow management engine converts the task of adding new predictive methods to the website to being a problem of configuration rather than one of writing new code. As such, adding new predictive methods to our website is now greatly streamlined. We expect addition of novel methods and updates to current existing methods will happen with increased regularity going forward. As the new backend makes all our methods available over REST we have also retired our older SOAP services. For further details of our workflow management engine we refer the reader to the Analytics Automated website (https://analyticsautomated.github.io).

## DISCUSSION

The latest update of the PSIPRED Workbench brings a whole suite of user centered improvements. These are designed to expedite the analyses users perform and layout the results in a more intuitive and accessible fashion. The PSIPRED Workbench has been in continuous operation for 20 years during which time there have been profound changes in the sizes of the biological databases and a wide proliferation of novel uses of algorithms and statistical methods throughout biology. The continued development of our server looks to ensure the PSIPRED Workbench is ready for the next 20 years of progress in the field of Bioinformatics.

## DATA AVAILABILITY

The PSIPRED Workbench is available via the following URL http://bioinf.cs.ucl.ac.uk/psipred.

## Supplementary Material

gkz297_Supplemental_File
