# Supplementary material for: The PSIPRED Protein Analysis Workbench: 20 years on
Source: Nucleic Acids Res. 2019 Apr 26;47(W1):W402–7. doi: 10.1093/nar/gkz297 (PMC6602445; doi:10.1093/nar/gkz297)
Supplement: gkz297_Supplemental_File [file gkz297_supplemental_file.pdf]

Supplementary Information

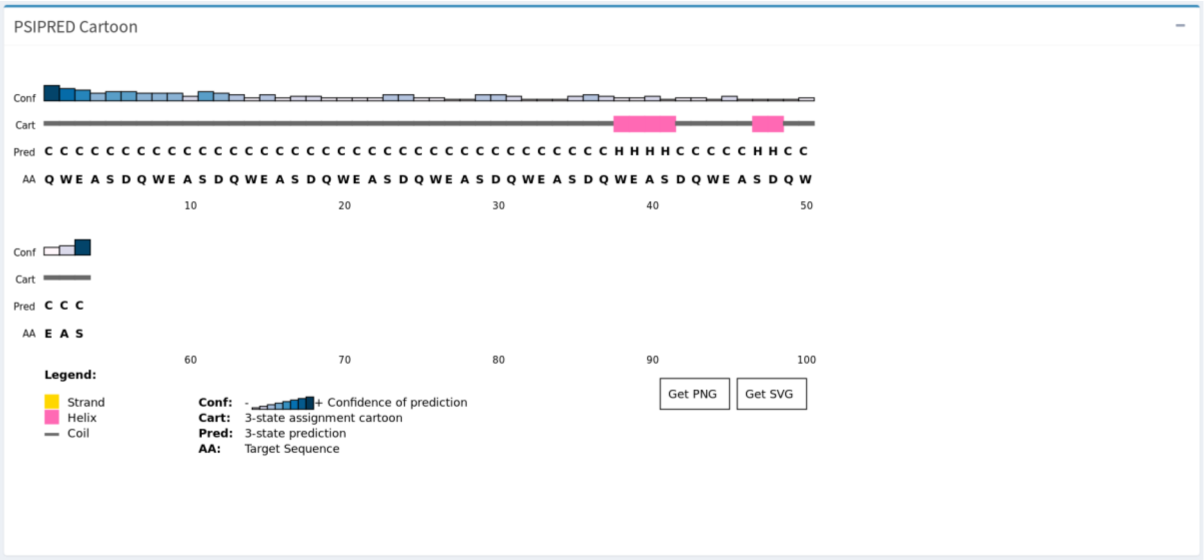

Figure 1: The PSIPRED results cartoon.

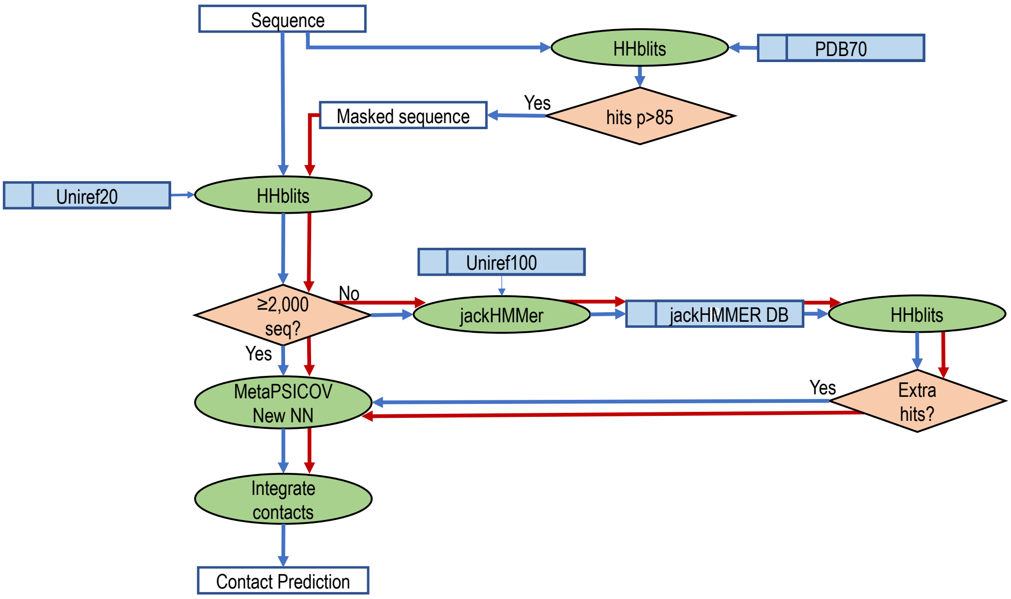

Figure 2: The flow diagram for MetaPSICOV2 data processing. The blue path is the path the input sequence takes. In the first branch HHBlits is used against the PDB sequences to attempt to detect domains or domain-like regions in the query sequence. In the second branch, if few sequences are detected, jackHMMer is used to attempt to enrich the alignment used for MetaPSICOV contact prediction. The red path shows the path followed by any domain-like regions, these have their own contacts predicted alongside the query sequence and these are integrated into the final prediction at the end.

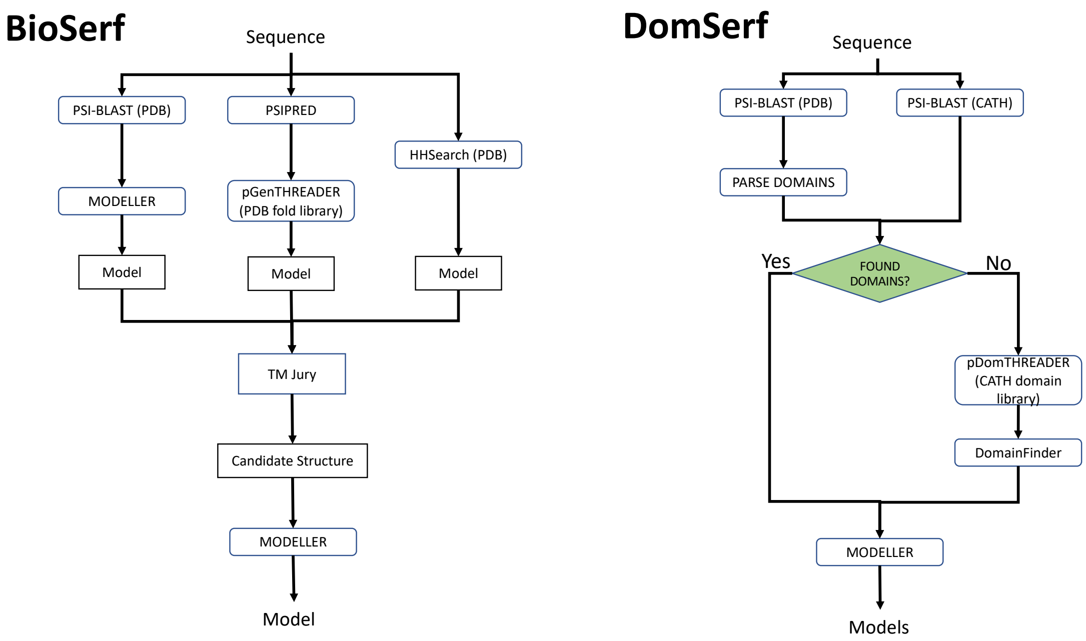

Figure 3: Pipelines for both DomSerf and BioSerf. The BioSerf pipeline makes three searches of the PDB using PSI-BLAST, pGenTHREADER and HHSearch. Each produces a set of models. The TMJury method then selects the cluster of most similar structures and these are used to generate a consensus model. The DomSerf method uses PSI-BLAST against the PDB and CATH database. Any whole PDB chain matches are parsed to divide the match in to domains. If the query sequence has sufficient coverage the match alignments are passed to MODELLER and the final models are produced. If the query sequence has little coverage pDomTHREADER is used to recognise CATH domains and any overlapping domains are resolved using DomainFinder. The set of best matching domains are then passed to MODELLER to produce the final models.
